# Heterogeneity in Cognitive and Socio-Emotional Functioning in Adolescents With On-Track and Delayed School Progression

**DOI:** 10.3389/fpsyg.2018.01572

**Published:** 2018-08-24

**Authors:** Loren Vandenbroucke, Wouter Weeda, Nikki Lee, Dieter Baeyens, Jon Westfall, Bernd Figner, Mariëtte Huizinga

**Affiliations:** ^1^Research Group of Parenting and Special Education, KU Leuven, Leuven, Belgium; ^2^Department of Methodology and Statistics, Leiden University, Leiden, Netherlands; ^3^Department of Clinical, Neuro- and Developmental Psychology, Vrije Universiteit Amsterdam, Amsterdam, Netherlands; ^4^Department of Counselor Education and Psychology, Delta State University, Cleveland, MS, United States; ^5^Behavioural Science Institute, Radboud University, Nijmegen, Netherlands; ^6^Department of Educational and Family Studies, Vrije Universiteit Amsterdam, Amsterdam, Netherlands

**Keywords:** graph theory, network analysis, community analysis, executive functioning, adolescence, cognitive development, social development, emotional development

## Abstract

Adolescence is characterized by considerable changes in cognitive and socio-emotional skills. There are considerable differences between adolescents with regards to the development of these skills. However, most studies examine adolescents’ average functioning, without taking into account this heterogeneity. The current study applies network analysis in order to examine heterogeneity of cognitive and socio-emotional functioning in adolescents on-track or delayed in their school progression. Data was collected at two time-points for on-track (*n* = 320) and delayed (*n* = 69) adolescents (*M*_age_ = 13.30 years, SD_age_ = 0.77). Repeated measures ANOVA showed no significant differences between the groups in cognitive and socio-emotional functioning (*p*’s > 0.05). Network analysis revealed that executive functions play a key role in the network of cognitive, social, and emotional functioning. This is especially the case in the delayed group where executive functions are even more central, both at T1 (inhibition and shifting) and T2 (shifting). Subsequent community analysis revealed three profiles in both groups: a well-adapted and well-balanced group, a group with high levels of need for arousal and risk-taking, and a group with regulation problems. Compared to on-track adolescents, delayed adolescents showed even higher levels of risk-taking in the second profile and higher levels of executive function problems in the third profile at T1. These differences were leveled out at T2, indicating adolescents in the delayed group catch up with their peers. This study highlights the intricate balance between cognitive, social and emotional functioning in adolescents in relation to school performance and provides preliminary evidence of the importance of taking individual differences within groups into account.

## Introduction

Adolescence is considered an important developmental stage (Konrad et al., 2013; Jaworska and MacQueen, 2015; Juraska and Willing, 2017). It is a period of transition that is not only characterized by obvious changes in biological and physical functioning, but also by rapid change in cognitive, social and emotional skills (Crosnoe and Johnson, 2011; Crone and Dahl, 2012). Adolescent thinking becomes more complex, abstract and focused on metacognition, while they reorient from the family to peers in the social domain and experience heightened intensity and variability of emotions in the emotional domain (Steinberg, 2005; Silvers et al., 2012; Blakemore and Mills, 2014; Dumontheil, 2014).

Due to the tremendous changes that occur during adolescence it is a period that offers both opportunities and challenges. For most adolescents, the increased cognitive, social and emotional abilities provide opportunities and result in increased competence, self-worth and positive social bonds (Briggs, 2009; Lewin-Bizan et al., 2010). Yet, some adolescents struggle with the many changes during this period and may become entangled in a negative spiral (Crone and Dahl, 2012). For example, adolescence is known to be accompanied by increases in anxiety, depression, risk-taking behavior, and academic problems (Prencipe et al., 2011; Eiland and Romeo, 2013; Blakemore and Mills, 2014; De Laet et al., 2016). Thus, there is great heterogeneity in the functioning of adolescents and the examination of these individual differences in adolescent developmental trajectories is essential to fully understand these cognitive, social and emotional changes. However, current studies often ignore the (neuropsychological) heterogeneity within samples. Additionally, most studies investigate cognitive, social and emotional skills in isolation, while in real life these skills are interrelated and influence each other. The current study attempts to fill these gaps by using network and community analyses to examine the interrelations between cognitive, social and emotional skills, while taking into account inter-individual differences between adolescents in their development.

### Cognitive, Social, and Emotional Changes in Adolescence

Three domains in which rapid and large developmental changes occur in parallel during adolescence are within cognitive, social and emotional functioning (Crone and Dahl, 2012). With regard to cognitive development, adolescents’ cognition becomes more complex and efficient and is characterized by increases in abstract, multidimensional and metacognitive thinking (Steinberg, 2005; Dumontheil, 2014). Central in this area of development are the substantial improvements with regard to executive functioning (Weil et al., 2013). These improvements can be seen both in increased performance on neuropsychological tasks measuring executive functioning (e.g., Digit Span, Go/No-go task; Zelazo and Carlson, 2012) and in increases of adolescents self-regulating behavior (e.g., reductions in hyperactivity; Coté et al., 2002). In the area of social development, adolescence marks a reorientation from family to peers (Blakemore and Mills, 2014). Adolescents spend more time with peers, interact with larger peer groups and interact more often with opposite-sex peers compared to children. As a consequence, peer problems are more likely to arise and peer influences on adolescents’ behavior become more apparent (Albert et al., 2013; Huang et al., 2014). Finally, changes in emotional functioning are characterized by the experience of more intense emotions (both negative and positive) and more variability in emotions, increases in need for arousal, and increases in emotional regulation abilities (Steinberg, 2005; Silvers et al., 2012).

### Interrelations Among Developmental Domains and Heterogeneity in Adolescence

Most available studies examining adolescent development and functioning study cognitive, social and emotional skills in isolation, while in real life these skills are known to influence each other. For example, the increase in emotion intensity and variability in combination with the slow and prolonged development of executive functions, can lead to a discrepancy between adolescents’ knowledge about negative consequences of behavior and their actual behavior in emotionally loaded situations, resulting in an increase in risk-taking behavior (Prencipe et al., 2011). Recent research even defines hot executive functions as the cognitive top-down control in emotionally important situations and distinguishes it from cool executive functions, which are used for top-down control in neutral (or purely cognitive) situations (Zelazo and Carlson, 2012). Similarly, due to the increased importance and influence of peers and the increased ability of metacognitive thinking, the way adolescents think about themselves and their emotional experiences (e.g., loneliness) changes (Blakemore and Mills, 2014; Vanhalst et al., 2014). These examples show that the general functioning of adolescents and their functioning at school is determined by the interrelation between cognitive, emotional and social skills, both at the neurocognitive level (e.g., executive functioning) and the behavioral level (e.g., risk-taking behavior). Yet, the interrelation between these skills is, so far, rarely systematically investigated. As a consequence, little is known about how these skills relate to each other, what the relative importance of each of these skills is, and what the role of a potential imbalance between these skills is for issues common during adolescence, such as academic difficulties.

Although the importance of individual variability in the functioning of adolescents is generally recognized, many studies do not explicitly examine this heterogeneity. Rather, most studies look at average development or functioning within samples or compare the average individual in one group with an average individual in another group. This is especially the case for community samples, which are often treated as a homogeneous group of individuals without symptoms or impairments (van der Meer et al., 2017). However, there is strong evidence that within a group of ‘typically developing’ individuals and adolescents there can also be large differences in cognitive, social and emotional skills (Fair et al., 2012; van der Meer et al., 2017). Not explicitly modeling or examining such heterogeneity can limit our understanding in the occurrence and causes of specific deficits (Fair et al., 2012).

### Grade Retention

One domain where great changes in cognition and socio-emotional functioning can result in maladaptive outcomes during adolescence is within education (De Laet et al., 2016). While obvious changes still have to occur in cognitive, social and emotional functioning, the altered school environment places high demands on these skills. For example, increases in class size, decreases in adult support and increased individual responsibility place higher demands on executive functioning and social skills (Jacobson et al., 2011). Consequently, difficulties with these skills can result in negative scholastic outcomes, such as grade retention. Previous research has shown behavior problems, aggressive behavior, peer relationships, motivation and self-efficacy, and executive functioning to be predictive for school outcomes such as drop out, grades and grade retention (Jimmerson et al., 2000; Robbins et al., 2004; Jimmerson and Ferguson, 2007; Davoudzadeh et al., 2015; Fitzapatrick et al., 2015).

Yet, current literature on grade retention focuses on examining the involvement of isolated aspects of cognitive, social and emotional functioning. Additionally, these studies often compare on-track and delayed adolescents, without taking the heterogeneity into account. This approach ignores that the balance between different skills can play a role in grade retention, and that different skills may have played a role in the retention of different adolescents. The current study examines the role of the interrelation between cognitive and socio-emotional skills in grade retention and takes into account the within group heterogeneity with the use of graph theory as an analytical approach.

### Graph Theory as an Analytical Approach

Graph theory involves the analysis of relationships within a network. For example, scores on measures of cognitive functioning can be represented as nodes within a network, and the correlations between these variables as edges which form the connections between the nodes. This approach has been applied in multiple domains, such as neuroimaging research examining connectivity between brain regions within neural networks (Dosenbach et al., 2013; Kellermann et al., 2015), or within the field of psychopathology when comparing patient and control groups (McNally, 2016). In the later it has, for example, been used to examine which symptoms (Heeren and McNally, 2016; Russell et al., 2017), which neuropsychological skills (Heeren and McNally, 2016; Hoorelbeke et al., 2016; Ibrahim et al., 2016) are central in clinical groups compared to non-clinical groups, or if core symptoms of clinical disorders vary across developmental periods (Martel et al., 2016).

Understanding which impairments are central for specific (clinical) groups can aid the (early) recognition and treatment of these problems (Ibrahim et al., 2016). This can be examined in more detail through detection of communities within graphs where individuals are represented as nodes, and the similarities between individuals (e.g., in their cognitive profiles) are represented by the edges. Communities are densely connected groups of nodes, with sparse connections to other groups. Individuals within a community show a high degree of similarity in the determinants of their behavior, while the various communities may display similar overt behaviors, but the underlying determinants often differ. One of the first studies to apply this method was conducted by Fair and colleagues (2012), who used community detection to determine the existence of subgroups based on neuropsychological profiles in both a typically developing group and an ADHD group of children and adolescents. Their results suggested that there was significant heterogeneity within both the patient and control groups, and that comparison between the groups was greatly improved when patients and controls were matched based on their neuropsychological profiles. Thus, the advantage of this approach is that rather than averaging out inter-individual differences in behavior, it enables examination of relative strengths and weaknesses within groups, and therefore can provide new information about underlying difficulties for specific groups.

### The Current Study

Where previous studies using graph theory as an analytical approach examined the networks of neuropsychological skills in clinical samples (e.g., ADHD), the current study shows that this approach can also be used to investigate the networks of skills in different developmental domains in a community sample of adolescents. The current study examines cognitive, social and emotional skills in adolescents on-track and delayed in their scholastic progress, adopting a graph theory approach. This approach allows examination and comparison of the influence of multiple cognitive (cool executive functioning, conduct problems, and hyperactivity), social (social support, resistance to peer influence, peer problems, and prosocial behavior) and emotional skills (emotional problems, emotion regulation, hot executive functioning, and need for arousal) in an on-track and delayed (in terms of their educational progress) adolescent group. With regard to cool executive functioning, the current study uses both performance tasks of working memory, inhibition and cognitive flexibility, as well as questionnaires assessing these and more complex executive functions (e.g., planning). This way the current study thoroughly examines three important core executive functions that still show clear developmental changes in early adolescence, as well as a broader perspective on executive functioning (Zelazo and Carlson, 2012; Diamond, 2013). Additionally, it allows the examination of the heterogeneity within these groups by investigating the existence of subgroups with specific patterns of cognitive, social and emotional skills within both the on-track and the delayed group. It is expected that network analyses can reveal differences between the on-track and delayed group of adolescents that are not apparent when applying traditional analytical approaches. Using this innovative approach allows for more nuanced insights into which skills play a role in grade retention for which subgroups of adolescents.

## Materials and Methods

### Participants

The present study used data from two waves. The target sample involved pre-adolescents and adolescents living in urban and rural areas in the Netherlands, attending regular schools for primary and secondary education. At baseline (T1), data were collected from 524 participants (mean age 13.13 years, SD = 0.86, 247 girls). Approximately 1 year later (mean time difference = 0.89 years; SD = 0.08), all participants were invited for data collection for the second time point (T2). A total of 101 participants moved, or indicated that they could not, or did not want to participate again. Therefore, at T2, data was collected from 423 participants (mean age 14.18 years, SD = 0.77, 199 girls). The children that dropped out between T1 and T2 differed slightly from the group that participated in both waves with respect to their age at T1 (*M_drop-out_* = 12.51, *M_both_* = 13.28; *t*(142.56) = 8.08; *p* < 0.001). This difference is strongly related to the younger children transferring to different schools for secondary education after middle school; we experienced great difficulty recruiting these children for participation in T2, as they failed to inform us about their new school. The children that dropped out did not differ from the children that participated in both waves with respect to their IQ at T1 [*M_drop-out_* = 25.86, *M_both_* = 25.39; *t*(171.30) = 1.28; *p* = 0.202], nor did they differ in terms of their gender distribution [*χ*^2^(1) = 0.00; *p* = 0.944] or the distribution of whether they repeated a class [*χ*^2^(1) = 1.08; *p* = 0.299].

A final sample of *N* = 389 (188 girls) was included in the analyses reported in the current study; these participants completed the entire task battery and all surveys in both waves (T1: mean age 13.30 years, SD = 0.77; T2: mean age 14.19 years, SD = 0.74).

Next, we identified the students who repeated a class during their school career (i.e., the delayed group, *n* = 69; 27 girls) as well as the students that never repeated a class (i.e., the on-track group, *n* = 320; 161 girls). The gender distributions in the delayed and the on-track group did not differ significantly, *χ*^2^(1) = 2.41, *p* = 0.120. At T1, the mean age in the delayed group slightly differed from the on-track group (13.81 years; SD = 0.79 vs. 13.19 years; SD = 0.72), *t*(93.01) = 6.00; *p* < 0.001. Consequently, at T2, the mean age in the delayed group slightly differed from the on-track group (4.72 years; SD = 0.76 vs. 14.08 years; SD = 0.69; *t*(92.85) = 6.36; *p* < 0.001). The delayed and the on-track group did not differ in terms of their IQ scores at T1 (*M_delayed_* = 25.13, *M_on-track_* = 25.43; *t*(106.08) = 0.66; *p* = 0.513) or at T2 (*M_delayed_* = 25.46, *M_on-track_* = 26.00; *t*(102.08) = 1.28; *p* = 0.203). Note that effects of age and IQ were regressed out in the analyses (see Results section for a description of the regression approach).

All participants provided written informed consent for the study (parental consent and participant assent for children and adolescents) at both time points. Instead of receiving individual credit, participants received a voucher for an excursion together with their classmates. The study was approved by the Ethical Committee of the Faculty of Behavioral and Social Sciences of the University of Amsterdam.

Estimated intelligence scores were obtained by using a non-verbal scale, the matrix reasoning subscale of the Stanford Binet V (Roid, 2003). At T1, the mean scores on this task were 25.13 (SD = 3.42) for the delayed group, and 25.43 (SD = 3.77) for the on-track group. At T2, the mean scores on this task were 25.46 (SD = 3.15) for the delayed group, and 25.98 (SD = 3.27) for the on-track group. Mean scores did not differ between waves [*F*(1, 387) = 2.04, *p* = 0.155], nor between groups [*F*(1, 387) = 1.41, *p* = 0.236].

### Materials

Participants’ cognitive, social and emotional functioning was indexed by surveys and behavioral measures. **Table 1** provides an overview of the measures and the variables of interest per domain of functioning.

**Table 1:** Overview of the measures and variables of interest per domain of functioning (i.e., cognitive, social, and emotional functioning).

Domain	Task or survey	Variables of interest
Cognitive functioning	Dots-Triangles Task Huizinga et al. (2006)	Ratio task-repeat/task-switch trials (RT)
	Eriksen Flankers Task Ridderinkhof and Van der Molen (1995); Huizinga et al. (2006)	Ratio congruent/incongruent trials (RT)
	Digit Span Kort et al. (2005)	Ratio digit span forward score/digit span backward score
	Columbia Card Task Figner et al. (2009)	Average of turned cards on cold version
	BRIEF Guy et al. (2004); Huizinga and Smidts (2012)	– Inhibit score
		– Shift score
		– Working memory score
		– Task completion score
		– Plan/organize score
		– Organization of materials score
		– Monitor score
	Strengths and Difficulties Questionnaire Goodman (1997, 2001); van Widenfelt et al. (2003)	– Conduct problems score
		– Hyperactivity/inattention score
Social functioning	Social Support Scale Harter (1985)	– Perceived social support from teachers score
		– Perceived social support from parents score
		– Perceived social support from classmates score
		– Perceived social support from close friends score
	Resistance to Peer Influence Steinberg and Monahan (2007)	Total score
	Strengths and Difficulties Questionnaire Goodman (1997, 2001); van Widenfelt et al. (2003)	– Prosocial behavior score
		– Peer problems score
Emotional functioning	Need for arousal Figner et al. (2009)	Total score
	Columbia Card Task Figner et al. (2009)	Average of turned cards on hot version
	Strengths and Difficulties Questionnaire Goodman (1997, 2001); van Widenfelt et al. (2003)	Emotional problems score
	BRIEF Guy et al. (2004); Huizinga and Smidts (2012)	Emotion regulation score

#### Surveys

##### SDQ

To assess child mental health problems, we used the Dutch self-report version of the Strengths and Difficulties Questionnaire (SDQ; Goodman, 1997, 2001; van Widenfelt et al., 2003). The SDQ consists of 25 items covering five subscales relating to emotional problems, peer relationship problems, conduct problems, hyperactivity/inattention and prosocial behavior. Each subscale comprises five questions with 3-point response scales (“Not true” = 0, “Somewhat true” = 1, and “Certainly true” = 2). Example items include “I am constantly fidgeting or squirming” (hyperactivity), or “I am kind to younger children” (prosocial behavior). The variables of interest were the mean scores per subscale, with higher scores indicating more mental health problems (A. Goodman et al., 2010).

##### RPI

To assess resistance to peer influence, we used an adapted version of the Resistance to Peer Influence questionnaire (Steinberg and Monahan, 2007). This adaptation consisted of ten statements, such as “My friends easily make me change my mind,” or “I say things I don’t really mean, when I think that my friends will respect me more.” Respondents indicated on 5-point response scales (“Not at all true” = 0, “Not true” = 1, “Somewhat true” = 2, “True” = 3, and “Certainly true” = 4) which of the answer options applied to them. The variable of interest was the total score of all items, with higher scores representing higher resistance to peer influence.

##### BRIEF

To assess daily life executive functions, we used the self-report version of the Behavior Rating Inventory of Executive Function (BRIEF; Guy et al., 2004; Huizinga and Smidts, 2012). The questionnaire was completed by the adolescents, who indicated how often a given behavior has occurred in the past 6 months by endorsing one of three responses, namely “Never,” “Sometimes,” or “Often.” The BRIEF consists of 75 items, concerning specific behaviors relating to executive functioning in children. The questionnaire comprises eight clinical scales (Inhibit, Shift, Emotional Control, Task Completion, Working Memory, Plan/Organize, Organization of Materials, and Monitor), used as variables of interest. Example items include: “Gets out of seat at the wrong times” (Inhibit), “Is disturbed by change of teacher or class” (Shift), or “Makes careless errors” (Monitor). Higher scores indicated more problems with executive functions.

##### Social support scale

To assess the perceived social support and regard which significant others manifest toward children and young adolescents, we used an adapted version of the Social Support Scale (Harter, 1985). This version consisted of 16 statements, concerning their parents (4 items), classmates (4 items), teachers (4 items), and close friends (4 items). Example items are: “I have parents who want to listen to my problems,” “I have classmates I can become friendly with,” “I have a teacher who cares about me,” or “I have a close friend who really understands me.” Respondents indicated on 5-point scales (“Not at all true” = 0, “Not true” = 1, “Somewhat true” = 2, “True” = 3, and “Certainly true” = 4) which of the answer options applied to them. The variables of interest were the total scores on the parents, classmates, teachers, and close friends scales. Higher scores indicated a higher perceived availability for social support.

##### Need for arousal

To assess situation-unspecific trait-like aspects of need for-arousal, we used an eight-item questionnaire devised by Figner et al. (2009). Questions refer to broad preferences regarding the level of novelty in general and the propensity to expose oneself to risky situations in everyday life (e.g., “I like a lot of variety” and “I often position myself in an exciting/dangerous situation on purpose”). Responses on this scale were given using a visual analog scale (slider bar), with scale endpoints “Does not apply at all” (scored as 1) and “Applies very much” (scored as 100). The variable of interest was the mean score of all eight questions.

#### Behavioral Measures

##### Dots-triangles

In order to assess cognitive flexibility, we used the Dots and Triangles task, which is part of a task battery to assess executive functions in children, adolescents and young adults (Huizinga et al., 2006). In a 4 × 4 grid on the screen, varying numbers (i.e., three to eight per half of the grid; equally distributed) of dots or triangles were presented. During the “dots” task, participants had to decide whether there are more dots in the left or the right part of the screen (block 1; 30 practice trials, 50 experimental trials). During the “triangles” task, participants had to decide whether there are more triangles in the top or in the bottom part of the screen (block 2; 30 practice trials, 50 experimental trials). Blocks 1 and 2 were administered in randomized order. In the third block (90 practice trials, 150 experimental trials), a series of four “dots” trials and a series of four “triangles” trials were alternately presented to the participants. The focus of the analyses was on reactions in the third block, in which responses could be preceded by a trial requiring the same task (i.e., task-repeat trials) and responses could require a switch to the alternative task (i.e., task-switch trials). Participants had 3500 ms to respond; when a response was given, the stimulus disappeared from the screen. The time interval between the response and the next stimulus varied pseudo-randomly between 900 and 1100 ms in steps of 10 ms. The main dependent variable was the ratio of the median response latencies on task-repeat trials and task-switch trials.

##### Eriksen flankers task

In order to assess the ability to resist interference, we used the Eriksen Flankers Task, which was also part of the task battery to assess executive functions in children, adolescents and young-adults (Ridderinkhof and Van der Molen, 1995; Huizinga et al., 2006). Participants had to respond to a left vs. right pointing arrow presented at the center of the screen, by pressing a left or right response button. The arrow was flanked by four arrows pointing in the same direction (i.e., →→→→→ or ←←←←←; congruent condition) or by four arrows pointing in the opposite direction (i.e., →→←→→ or ←←→←←; incongruent condition). There were 50 practice trials and 100 experimental trials (i.e., 50 congruent trials and 50 incongruent trials, varied pseudo-randomly). The stimulus onset occurred with the presentation of a rectangle, which served as the warning stimulus. After a time interval of 400 to 600 ms (varied pseudo-randomly in steps op 10 ms), the arrows array was presented. Participants had 2500 ms to respond; the arrow array disappeared from the screen when a response was made. The inter trail interval varied pseudo-randomly between 900 and 1100 ms in steps of 10 ms. The main dependent variable was the ratio of the median response latencies on congruent trials and incongruent trials.

##### Digit span

To asses working memory capacity, we used a computerized version of the Digit Span subtest from the WISC-III-NL (Kort et al., 2005). The Digit Span comprises two parts: the Digit Span Forward and the Digit Span Backward. The Digit Span Forward requires the participant to repeat increasingly longer strings of numbers, in the same order as presented on the computer screen. The Digit Span Backward requires the participant to repeat increasingly longer strings of numbers, in the reverse order as presented on the computer screen. Numbers were presented at a rate of one number per second, and responses were given on a number pad. In both parts of the Digit Span, each test item consisted of two strings of digits administered at each list length, starting with a string of two digits, and increasing in length by one digit following successful repetition of at least one string of digits at a given length. Testing was discontinued when a participant incorrectly repeated two strings of the same length. Digit Span scores were computed using the longest string length correctly recalled. The variable of interest was the ratio of the Digit Span Forward and the Digit Span Backward.

##### Columbia card task

In order to assess risk-taking behavior, we used a computerized version of the Columbia Card Task (CCT; Figner et al., 2009). The CCT comprises a hot and a cold version. The hot version of the CCT was designed to trigger the involvement of affective decision-making processes, whereas the cold version of the CCT was designed to assess risk-taking under predominantly deliberative conditions involving ‘cold’ cognitive processes. In the hot version, 32 cards are presented face down in an 8 × 4 grid on the screen (see Figner et al., 2009 for an example of the lay-out). Among these cards are loss cards and gain cards. At the top of the screen, information is provided about the number of loss cards (1 or 3) hidden among them, the gain amount for each turned over gain card (10 or 30 points), and the loss amount when a loss card is turned over (−250 or −750). There were 24 game rounds. Each new game round starts with a score of 0 points and all 32 cards shown face down. Within a game round, participants are required to make a series of binary decisions whether to turn over a card or to stop turning over cards. After each turn, they receive feedback, indicating whether the turned card was a gain card or a loss card. A running total of the accumulated amount of points is shown when a card is turned over. A game round continues until the participant turns over a loss card (leading to the subtraction of the loss from the running score), or when the participant decides to stop tuning cards. The magnitude of gain, magnitude of loss, and the number of loss cards varied across game rounds. The variable of interest is the average number of cards chosen to turn, as an indicator of risk-taking. The cold version of the CCT is similar to the hot version, but without the inclusion of affective processes during decision making. Specifically, at the beginning of each game round the participant decides how many cards he or she wants to turn over. In addition, outcome feedback is only provided until all game rounds have been played. Again, the variable of interest is the average number of cards chosen to turn.

### Procedure

Testing took pace in two sessions. In one session, the experimental tasks were administered. All participants were tested simultaneously in groups of two. The order of the tasks was counterbalanced. There were 3-min breaks between tasks. Each test was practiced first; when the participants understood the instructions, the actual testing took place. Each experimental test session test session lasted approximately 1 h. In the other test session, participants filled out the surveys. This was done in groups of approximately 15 participants, and took place in the common computer lab at school, which was reserved for testing. Each survey testing session lasted approximately 1 h.

## Results

We performed three sets of analyses. The first set included analyses of variance to assess performance differences between the delayed and the on-track group, and between waves. Data are presented in three domains: cognitive functioning, social functioning and emotional functioning (see 2). Age and IQ were used as covariates. The second set included a network analysis to model pairwise relationships between the variables in the delayed and the on-track groups. The third set included a community profile analysis to assess neuropsychological heterogeneity in relation to educational performance (i.e., delayed or on-track). To control for differences in Age and IQ in the network analysis we fitted, for each variable in each wave, a linear model with Age and IQ as predictors and the variable of interest as dependent variable. The residuals of each regression were subsequently used to calculate the covariance matrix used as input in the network analysis. Analyses were performed using the *lm* function in R (R Core Team, 2016).

### Analysis of Variance

The marginal means and standard errors of the variables of interest per domain of functioning and per time point (T1 and T2) are reported in **Table 2** for the delayed and on-track group.

**Table 2:** The interaction effects of the variables of Interest (per domain of functioning) at T1 and T2, with group (delayed vs. on-track).

Domain/variable of interest	Delayed group (*n* = 69)		On-track group (*n* = 320)		*F*	*p*
	T1 M (SD)	T2 M (SD)	T1 M (SD)	T2 M (SD)		
**Cognitive functioning**						
Dots-Triangles	1.26 (0.18)	1.28 (0.23)	1.28 (18)	1.28 (0.17)	0.185	0.668
Eriksen Flankers Task	1.13 (0.06)	1.13 (0.06)	1.13 (0.06)	1.13 (0.05)	0.000	0.997
Digit Span	0.77 (0.30)	0.81 (0.40)	0.92 (0.62)	0.81 (0.25)	2.925	0.088
Columbia Card Task (cold version)	3.14 (4.68)	1.68 (3.83)	3.21 (4.19)	1.61 (3.64)	0.050	0.824
BRIEF Inhibit	1.55 (0.40)	1.57 (0.39)	1.53 (0.34)	1.55 (0.35)	0.003	0.957
BRIEF Shift	1.47 (0.40)	1.43 (0.37)	1.41 (0.30)	1.41 (0.35)	0.853	0.356
BRIEF Working Memory	1.56 (0.39)	1.56 (0.36)	1.58 (0.37)	1.60 (0.38)	0.080	0.777
BRIEF Task Completion	1.55 (0.45)	1.55 (0.40)	1.50 (0.36)	1.53 (0.38)	0.173	0.678
BRIEF Plan/Organize	1.53 (0.39)	1.52 (0.34)	1.52 (0.34)	1.54 (0.36)	0.486	0.486
BRIEF Organization of Materials	1.53 (0.40)	1.50 (0.38)	1.58 (0.41)	1.61 (0.43)	1.589	0.208
BRIEF Monitor	1.51 (0.46)	1.48 (0.43)	1.53 (0.44)	1.51 (0.42)	0.001	0.976
SDQ Conduct problems	1.93 (1.83)	1.93 (1.63)	1.61 (1.37)	1.64 (1.33)	0.021	0.884
SDQ Hyperactivity/inattention	3.13 (1.96)	3.20 (1.97)	3.14 (2.03)	3.19 (1.99)	0.012	0.913
**Social functioning**						
Social Support Scale – Parents	4.52 (0.74)	4.52 (0.73)	4.59 (0.62)	4.61 (0.63)	0.066	0.797
Social Support Scale – Classmates	3.48 (0.59)	3.38 (0.63)	3.47 (0.53)	3.51 (0.54)	2.317	0.129
Social Support Scale – Teachers	3.23 (1.07)	3.24 (1.02)	3.37 (0.95)	3.11 (0.98)	2.917	0.088
Social Support Scale – Close friends	4.39 (0.77)	4.46 (0.79)	4.29 (0.85)	4.39 (0.77)	0.076	0.783
Resistance to peer influence	2.17 (0.45)	2.12 (0.59)	2.26 (0.50)	2.16 (0.52)	0.689	0.407
SDQ Prosocial behavior	3.17 (1.15)	2.94 (1.26)	7.91 (1.73)	7.79 (1.86)	0.002	0.961
SDQ Peer relationship problems	7.91 (1.45)	7.61 (1.97)	2.96 (1.33)	2.72 (1.18)	0.651	0.420
**Emotional functioning**						
Need for arousal	48.37 (12.50)	48.78 (12.91)	50.57 (12.47)	51.18 (13.36)	0.013	0.909
Columbia Card Task (hot version)	4.07 (2.46)	2.75 (2.45)	3.55 (2.64)	2.51 (2.62)	0.598	0.440
SDQ Emotional symptoms	2.10 (1.84)	2.01 (1.98)	2.36 (1.80)	2.19 (1.95)	0.121	0.728
BRIEF Emotional Control	1.29 (0.33)	1.23 (0.29)	1.32 (0.31)	1.32 (0.32)	2.277	0.132

*NB. df = 1,387.*

We performed separate repeated-measures ANOVA’s with Time Point (T1 and T2) as within-subject variable, and Group (delayed and on-track) as between-subjects variable. As can be seen in **Table 2**, none of the interactions were significant, indicating that the combined effect of Time point and Group on the variables of interest was absent. In other words, on average both groups showed the same change in scores from T1 to T2 on all measures.

### Network Analysis

Network analysis was performed on the covariance matrix of the 24 pre-processed variables. To estimate the optimal network in each group we used the GLASSO algorithm (Friedman et al., 2008) with an Extended BIC (EBIC, Foygel and Drton, 2010) criterion to select the optimal sparseness parameter. We estimated the optimal model on the covariance matrix of participants of both groups, but separately for each wave. Using this sparseness parameter, we then estimated the network separately for the on-track and delayed group in each wave. After network estimation, we calculated degree centrality for each node. To obtain confidence intervals we used a bootstrap approach with 1000 iterations. For each iteration, we took a random sample (with replacement) from each group and performed sparseness parameter selection (over both groups) and network estimation. All analyses were performed using the *bootnet* package (Epskamp et al., 2016) in R (R Core Team, 2016).

**FIGURE 1:**
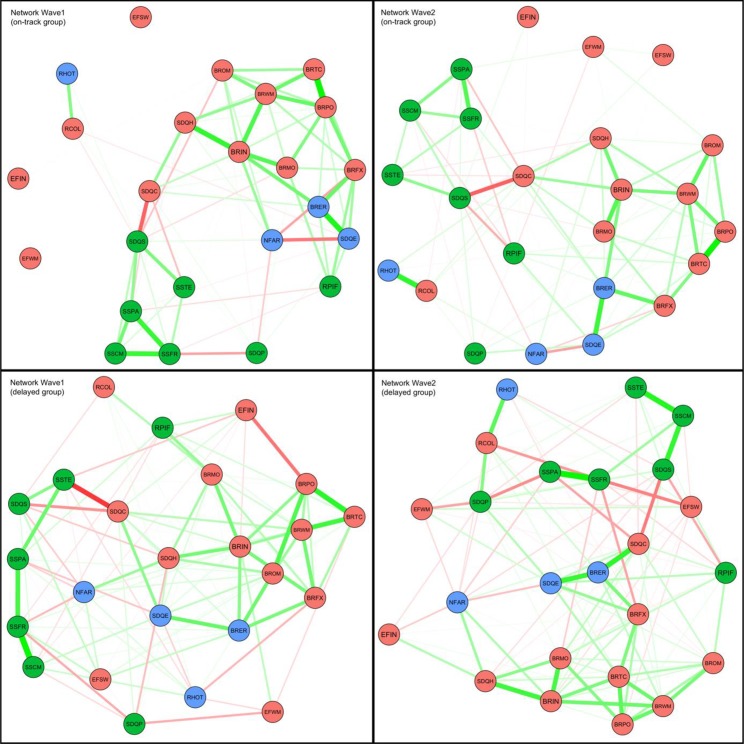
Network model of all variables, for the two waves across the three domains (cognitive functioning in red; social functioning in green; and emotional functioning in blue) and groups (delayed vs. on-track). The variables are now plotted in a ‘spring’ plot (Fruchterman and Reingold, 1991), in which variables that are strongly related are closer together and the more central nodes are in the center of the plot. EFSW, Dots-Triangles; EFIN, Eriksen Flankers Task; EFWM, Digit Span; RCOL, Columbia Card Task (cold version); BRIN, BRIEF Inhibit; BRFX, BRIEF Shift; BRWM, BRIEF Working Memory; BRTC, BRIEF Task Completion; BRPO, BRIEF Plan/Organize; BROM, BRIEF Organization of Materials; BRMO, BRIEF Monitor; SDQC, SDQ Conduct problems; SDQH, SDQ Hyperactivity/inattention; SSPA, Social Support Scale – Parents; SSCM, Social Support Scale – Classmates; SSTE, Social Support Scale – Teachers; SSFR, Social Support Scale – Close friends; RPIF, Resistance to peer influence; SDQS, SDQ Prosocial behavior; SDQP, SDQ Peer relationship problems; NFAR, Need for arousal; RHOT, Columbia Card Task (hot version); SDQE, SDQ Emotional symptoms; BRER, BRIEF Emotional Control.

**FIGURE 2:**
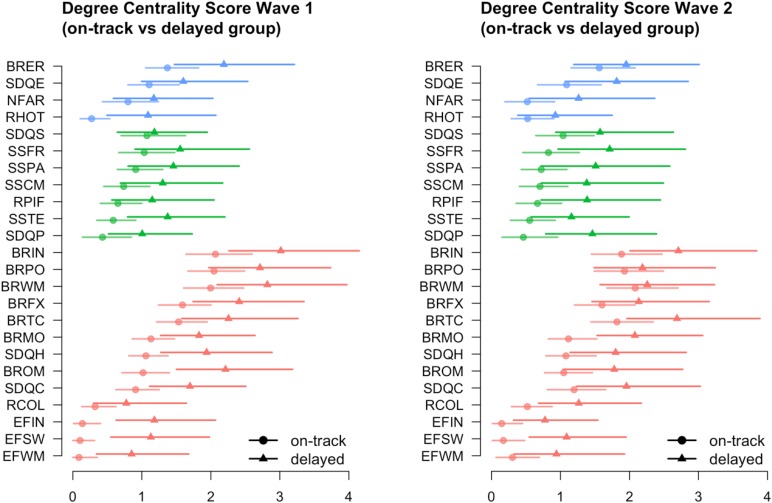
The centrality estimates and 95% confidence intervals of all variables, for the two waves across the three domains (cognitive functioning in red; social functioning in green; and emotional functioning in blue) and groups (delayed [triangles] vs. on-track [circles]). EFSW, Dots-Triangles; EFIN, Eriksen Flankers Task; EFWM, Digit Span; RCOL, Columbia Card Task (cold version); BRIN, BRIEF Inhibit; BRFX, BRIEF Shift; BRWM, BRIEF Working Memory; BRTC, BRIEF Task Completion; BRPO, BRIEF Plan/Organize; BROM, BRIEF Organization of Materials; BRMO, BRIEF Monitor; SDQC, SDQ Conduct problems; SDQH, SDQ Hyperactivity/inattention; SSPA, Social Support Scale – Parents; SSCM, Social Support Scale – Classmates; SSTE, Social Support Scale – Teachers; SSFR, Social Support Scale – Close friends; RPIF, Resistance to peer influence; SDQS, SDQ Prosocial behavior; SDQP, SDQ Peer relationship problems; NFAR, Need for arousal; RHOT, Columbia Card Task (hot version); SDQE, SDQ Emotional symptoms; BRER, BRIEF Emotional Control.

Estimated networks for the on-track and delayed groups for both waves are shown in **Figure 1**. Colored circles indicate the nodes of the network (red = cognitive functioning, green = social functioning, and blue = emotional functioning). Lines between the nodes are the non-zero partial correlations (red = negative and green = positive), thickness of the lines indicates stronger correlations. In order to examine differences between the on-track and delayed group, we plotted so-called ‘spring’ plots (Fruchterman and Reingold, 1991), in which variables that are strongly related are plotted closer together and more central (i.e., in the center) of the plot. In addition, **Figure 2** shows the centrality estimates for all variables for the two waves across the three domains.

As can be seen in **Figure 1**, across all waves and groups there are strong connections within the cognitive domain, indicating that the variables comprising this domain are strongly related. For the other domains, the interrelations are present, but less strong. Across domains of functioning, there are strong connections of executive function behavior (as measured by questionnaires) and emotion regulation, and of conduct problems and prosocial behavior and teacher social support. Overall, the networks for both groups are relatively stable across waves.

The most prominent difference between the delayed and the on-track group is expressed in the increased amount of connections within and between social and emotional variables in the delayed group compared to the on-track group. Remarkably, in the delayed group, the variables in the emotional domain are more central than in the on-track group. Looking at the individual variables per domain (**Figure 2**), in the cognitive functioning domain, hyperactivity (SDQ), conduct problems (SDQ), inhibition, shifting, monitoring, and difficulties with organization of materials (BRIEF) play a more central role in the delayed group compared to the on-track group. In the social functioning domain, only teacher social support seems to play a more prominent role in the delayed group than in the on-track group. Whereas in the emotional functioning domain, especially risk-taking in the hot condition plays a more central role in the delayed group compared to the on-track group. The observed differences in all three domains become smaller in the second wave.

### Community Profile Analysis

Community profile analysis was performed using an approach similar to that of Fair and colleagues (2012). We first transformed all variables for both groups and waves to *z*-scores. To aid interpretability of the profiles we clustered related variables using Confirmatory Factor Analysis (CFA, using the Lavaan package in R, R Core Team, 2016) separately for each wave. For an overview of the factors see **Table 3**. The resulting factor scores for each participant indicate their individual profile. We then correlated each participant’s profile with the profiles of all other participants. On this 389 × 389 correlation matrix, we performed a community analysis using the Louvain algorithm (Rubinov and Sporns, 2011) in R. We ran the analysis 200 times and further examined the analysis with the highest modularity index [Q, higher index indicates better separable ‘modules’ or ‘communities,’ ranges from −0.5 to 1 with positive scores indicating above chance level separation in modules (Newman, 2004)]. In addition, we examined the uniqueness of the communities (i.e., a significant variation from random). We compared our Q index against an estimated distribution of Q values under the null hypothesis (Fair et al., 2012). For this approach, we randomized the factor scores of all participants (separately for each wave) 200 times and estimated the Q index at each instance. The distribution of Q’s across the 200 iterations was taken as our null distribution to which we compared our observed Q values for each wave.

**Table 3:** Overview of profiles for community profile analysis.

Factor	Name (abbreviated)	Items in factor
**Cognitive functioning**		
Executive Functioning Tasks	EF Task	Dots-Triangles, Eriksen Flankers Task, and Digit Span
Risk-taking (no affect)	Risk Cold	Columbia Card Task (cold version)
Executive Functioning Behavior	EF Daily	BRIEF – Inhibit, Shift, Working Memory, Task Completion, Plan/Organize, Organization of Materials, and Monitor
Behavior regulation problems	Reg Prob	SDQ – Conduct problems, Hyperactivity/inattention
**Social functioning**		
Social support	Soc Supp	Social Support Scale – Parents, Classmates, Teachers, and Close friends
Resistance to peer influence	RPI	Resistance to peer influence
Prosocial behavior	Pro Soc	SDQ Prosocial behavior
Peer problems	Peer Prob	SDQ Peer relationship problems
**Emotional functioning**		
Need for arousal	NFA	Need for arousal
Risk-taking (affective)	Risk Hot	Columbia Card Task (hot version)
Emotional problems	Emo Prob	SDQ Emotional symptoms
Emotion regulation	Emo Reg	BRIEF Emotional Control

The community analysis thus estimates a number of profiles, with the number of profiles decided by the algorithm, that best separates participants into different ‘profile communities’ and assigns each participant to one community. We analyzed the resulting profiles in terms of mean factor scores for the twelve factors, differences in these scores across the on-track and delayed groups, and the proportion of participants across both groups assigned to a certain community.

**FIGURE 3:**
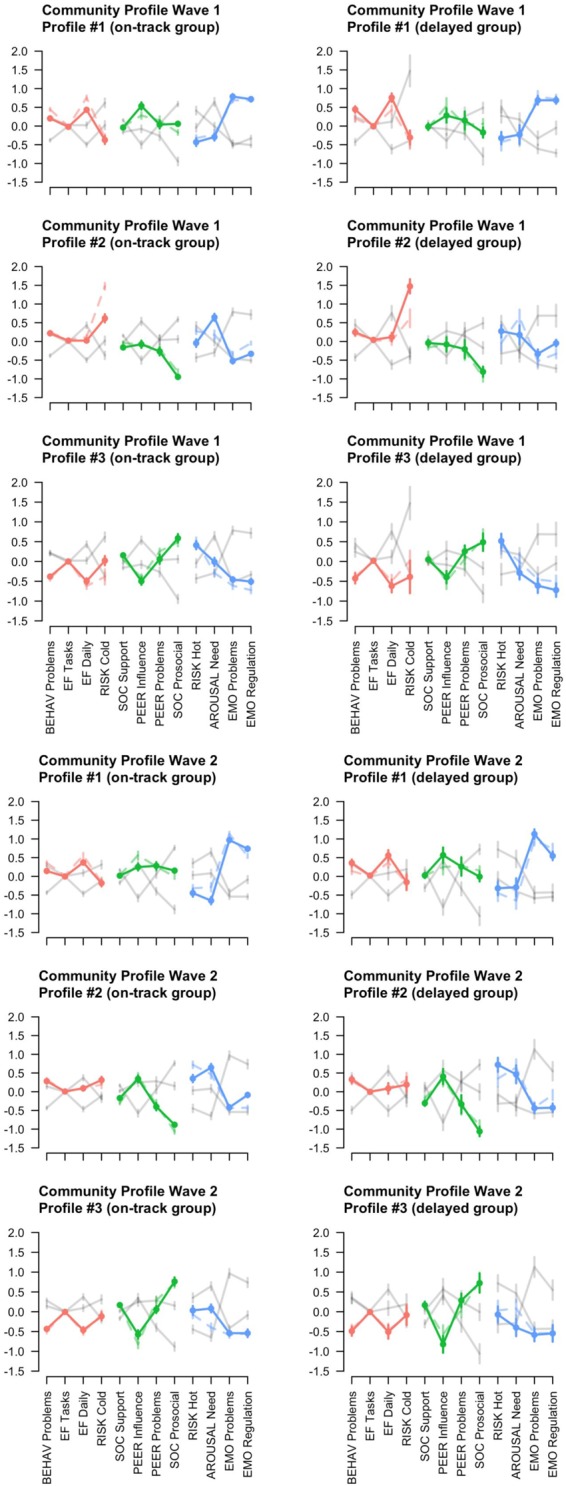
The three profiles that resulted from the community analysis, per wave. Profile #1 is an ‘EF problems’ profile; Profile #2 is a ‘high risk-taking behavior’ profile; Profile #3 is profile without significant behavioral problems. Colors indicate the specific domains (red, Cognitive; green, Social; and blue, Emotional). For comparison, the dotted lines indicate the ‘opposite group,’ i.e., in the on-track profiles the dotted line indicates delayed profile and vice-versa. The gray lines indicate the ‘opposite profile’ per group, i.e., the gray lines in the profile #1 plot indicate the profiles #2 and #3.

The community analysis returned three profiles for both waves with a good modularity index (Q T1 = 0.42 and Q T2 = 0.45) (Newman, 2004, 2006). These Q values differed significantly from our estimated null distributions (*z*-values 10.05, and 16.10 for T1 and T2, respectively), indicating that all profiles are unique (i.e., they vary significantly from random). **Figure 3** shows the profiles, whereas **Table 4** shows the distribution of scores across the profiles. Profile #1 could be interpreted as a ‘weak executive function and emotion regulation profile,’ as shown by more problems with executive functions, more prone to influence of peers, relatively normal problems with peers, low risk-taking, but more emotion regulation problems. This profile is somewhat more inflated in the delayed group compared to the on-track group. Profile #2 seems to be characterized by high risk-taking behavior, in both the affective (hot) and non-affective (cold) setting, with a high need for arousal. Especially cold risk-taking is at higher level in the delayed group compared to the on-track group, while for hot risk-taking this is effect is reversed. Profile #3 seems to be a relatively balanced profile, with the absence of problems with EF, influence by peers, and peer problems, high levels of prosocial behavior, a somewhat high need for arousal, and no problems with emotion regulation. At T2 all profiles look very similar except the high cold risk-taking of profile 2 is now less extreme.

**Table 4:** Proportion of participants in each profile per group and wave.

	On-track	Delayed
**Wave 1 (*Q* = 0.42)**		
Profile 1	40.3	33.3
Profile 2	25.3	15.9
Profile 3	34.4	50.7
**Wave 2 (*Q* = 0.45)**		
Profile 1	33.1	31.9
Profile 2	32.8	40.6
Profile 3	34.1	27.5

*Q, modularity strength. Higher index indicates better separable ‘modules’ or ‘communities,’ ranges from −0.5 to 1 with positive scores indicating above chance level separation in modules (Newman, 2004).*

## Discussion

Although it is generally accepted that adolescence is characterized by high levels of neuropsychological heterogeneity, traditional statistical analyses fall short when taking into account the interrelation between different characteristics of interest and the relation between these profiles and (sub)group membership. Building on recent insights from research on neural networks (e.g., Kellermann et al., 2015) and social networks (e.g., Cappella et al., 2013), this study introduced network analysis as a statistical approach to better understand the interrelation between aspects of cognitive, social and emotional functioning in typically developing adolescents who are either on-track or delayed in their school career. In a next step, community analysis was used to assess neuropsychological heterogeneity in relation to educational performance. It was expected that this network approach could reveal differences between on-track and delayed students that would not be visible using traditional analytical approaches. Results indicate that traditional analysis of variance does not reveal significant differences between the on-track and the delayed group. In contrast, network analysis showed heightened centrality of executive functions in the delayed group and community analysis indicated increased levels of risk-taking and executive function problems to be important for adolescents delayed in their school career. This provides a first indication of the importance of examining heterogeneity and interrelations among different developmental domains in order to understand adolescent functioning and school retention.

First, the on-track and delayed group were compared using traditional analyses, namely repeated measures ANOVA. The on-track and delayed group were followed up for approximately 1 year to map their development in terms of the interrelation of cognitive, social and emotional functioning. Repeated measures ANOVA’s showed that for each of the variables, namely executive functions, risk-taking (no affect), behavior regulation problems, social support, resistance to peer influence, prosocial behavior, peer problems, need for arousal, risk-taking (affective), emotional problems, and emotion regulation, the change from T1 to T2 was identical for the on-track and the delayed group.

This is in contrast to previous studies that found a relationship between different cognitive, social and emotional skills and school outcomes such as grade retention (Jimmerson et al., 2000; Robbins et al., 2004; Jimmerson and Ferguson, 2007; Davoudzadeh et al., 2015; Fitzapatrick et al., 2015). For example, Jimmerson and Ferguson (2007) found grade retention to have a negative impact in the sense that children who were retained in the beginning of elementary school showed more aggressive behavior in adolescence. However, the latter study specifically focused on children experiencing delay early in their school career and the long term consequences of this delay, while the current study did not specify the timing of the retention. It is possible that early retention has a more profound impact on children, that there are different causes for early and late grade retention, or that deficits will become more visible over a longer period of time in adolescents’ development. Additionally, many studies examining cognitive, social and emotional functioning in relation to grade retention have been conducted in the United States, where school policies and policies on grade retention are likely different compared to the Netherlands. Whereas grade retention in the United States is often based on results on standardized tests (Jimmerson and Ferguson, 2007), the decision to retain a child or youth in the Netherlands is made by the teachers and based on a multitude of factors, such as grades and behavior. As the motives for grade retention are different, the population of delayed adolescents is also likely to differ, making the comparison of existing insights more difficult.

Nevertheless, subtle differences can exist between the two groups of adolescents which are not detected by these traditional analyses. Therefore, network analysis was used to examine the interrelation between the different variables of cognitive, social and emotional functioning for the on-track and the delayed group. Network analyses allow for the examination of the interrelation between different variables and shows which variables are very central (i.e., strongly related to many other variables) or peripheral (i.e., weakly related to the other variables). Networks of the on-track group suggest that executive functions as measured by cognitive tasks, are peripheral in the network and thus play a limited role in the cognitive, social and emotional functioning of adolescents. Yet, difficulties with self-reported executive function behavior, as measured with a questionnaire, play a more central role in the network. In the delayed group, however, only self-reported executive function behavior stands out in the network, playing a central role, while all other variables, including executive function as measured with cognitive tasks, are equally important in the network. The comparison of both groups shows that at T1 the cognitive measures, teacher support, and risk-taking in affective situations are more central in the delayed adolescents compared to the on-track adolescents. Especially executive functions (inhibition, shifting, and difficulties with organization of materials) play a more central role in the functioning of delayed adolescents compared to the on-track adolescents. At T2 these differences have diminished and only shifting is significantly more central in the network of delayed adolescents compared to on-track adolescents. In other words, while traditional analysis does not show any differences between the two groups, network analysis suggests that executive functions are more important in the functioning of delayed adolescents than they are for on-track adolescents.

The centrality of executive function behavior in the network indicates that this skill is strongly related to many other skills of adolescents cognitive, social and emotional functioning. This is perhaps not surprising as executive functions are crucial for goal-directed behavior (Huizinga et al., 2006). Executive functioning has been related to a large number of outcomes in a variety of live domains including physical health, mental health, and school functioning (Best et al., 2011; Moffitt et al., 2011; Diamond, 2013). Adding to existing literature, the current study suggests that for adolescents delayed in their scholastic track, executive function behavior may be even more strongly related to a multitude of other domains and outcomes. Also, while the heightened importance of executive functions for delayed adolescents was clear at the first wave, this importance diminished to some extent by the second wave of data collection. It is possible that adolescents who are retained end up in a more appropriate age group, which can benefit the development of certain skills and lead to a more balanced network of cognitive, social and emotional skills.

Third, community analyses were conducted to assess neuropsychological heterogeneity within the two groups. Three unique profiles were found within both groups and at both waves of data collection. The first profile comprises adolescents with difficulties in emotion regulation, self-reported executive function behavior, and problems with resistance to peers, while showing low levels of risk-taking, and low levels of need for arousal. The second profile consists of adolescents with low levels of prosocial behavior, and high levels of risk-taking behavior and need for arousal. Finally, the third profile includes a well-adapted group of adolescents, where most variables center around the average. This group indicates low levels of difficulties in executive function behavior, high levels of prosocial behavior, and average levels of resistance to peer influence. When comparing the on-track and delayed group, the well-adapted profile shows few differences. However, in the first profile delayed adolescents show a slightly higher level of difficulties in executive function behavior. In the second profile the delayed adolescents show even higher levels of risk-taking behavior in non-affective settings. These differences are visible at T1, but again are leveled off at T2. This suggests that while some delayed adolescence may show more unbalanced profiles, they eventually catch up with their on-track counterparts.

An important note is warranted on the distribution of the delayed and on-track adolescents across the different profiles. Results show that at T1 half of the delayed adolescents are classified in the balanced group (profile 3) and few are categorized in the high risk taking group (profile 2). By the second wave of data collection these numbers reverse and more delayed adolescents are categorized in the risk-taking profile. For the on-track adolescents, the distribution across the three profiles is more equal. It would be expected that the delayed adolescents are more often categorized in the unbalanced profiles. One possible explanation is that the current study may not have included all skills relevant for grade retention, and therefore misses additional profiles of adolescents. Another explanation is that, as mentioned before, children and youth in the Netherlands are often retained to prevent them to change into a lower academic track. Such a preventive approach may result in retaining some adolescents who have few deficits. The fact that more delayed adolescents move to the unbalanced profiles may indicate that this approach has a negative effect for these balanced adolescents.

The findings of these community analyses thus further nuance the results of the network analysis and indicate that while executive functioning is important in the development of the delayed group as a whole, there are individual differences within this group. Whereas for some delayed adolescents executive functioning (behavior) plays an important role in their development, risk-taking might be the central deficit for others. As the network analyses in this study point out the centrality of executive function behavior in adolescents functioning, this may suggest that especially the group with difficulties in this executive function behavior are at risk for developing a multitude of problems. This has important implications for future research and clinical and educational practice. The results of the current study suggest that caution is warranted when average effects or group differences are examined. Such approaches may miss important associations and effects or even lead to wrong conclusions. An approach taking into account heterogeneity provides more detailed insights which can also be used to design more effective measures to reduce deficits. The current study for example indicates that while reducing deficits in executive functioning might be a useful intervention to reduce academic difficulties for some adolescents, this method will not be helpful for others.

### Strengths and Limitations

A clear strength of the current study is the advanced analytical approach used to examine different domains of adolescents functioning (e.g., executive functioning) in relation to grade retention. This provides us with new and more nuanced insights into these important topics in education. Additionally, community analyses can provide a powerful means of answering research questions, as it allows for new types of research questions to be examined (e.g., about heterogeneity) as well as avoiding problems generally linked to traditional analyses (e.g., multiple comparison in ANOVA).

Naturally, the current study also has some limitations, which should be taken into account. The current study used a large sample of adolescents, but only 69 of them belonged to the delayed group. Non-parametric procedures with bootstrapping were used to allow community analyses to be performed. Nevertheless, sampling bias occurs more easily in smaller samples, which means that the results of the current study might be biased due to an overrepresentation of certain (unknown) characteristics. Future studies should attempt to examine similar processes either in larger samples of adolescents who experienced grade retention or use a careful recruitment procedure that ensures that the sample is representative of the population with regard to key characteristics. Larger groups might also allow to distinguish more rare profiles that could not be found in the current sample. Additionally, it would be informative to examine heterogeneity within delayed and on-track groups, and interrelations between domains of functioning across a longer period of time. This could provide insights into developmental trajectories of different subgroups of adolescents.

## Conclusion

Traditional analyses often examine different aspects of adolescent cognitive, social and emotional functioning in isolation and rarely take into account the heterogeneity within groups of adolescents. The current study shows that such an approach may miss important insights. In the current study, no differences were found between on-track and delayed adolescents using traditional analysis of variance, while network analysis highlighted the importance of executive function behavior. Additionally, community analysis suggested executive function problems and risk-taking behavior to be important deficits for different subgroups of delayed adolescents. Network and community analyses can thus provide more nuanced insights into the underlying factors of specific difficulties, such as difficulties in educational progression. Such nuanced insights can guide more effective preventive and supportive measures for educational difficulties.

## Author Contributions

LV wrote the Introduction and Discussion of the manuscript, contributed substantially to the interpretation of the analyses, and reviewed the manuscript. WW contributed substantially to the design of the work, conducted and interpreted the network and community analyses, contributed substantially to the writing (Results section) and reviewing of the manuscript. NL contributed substantially to the design of the work, the interpretation of the analyses and the writing (Introduction) and reviewing of the manuscript. DB contributed substantially to the interpretation of the analyses and the reviewing of the manuscript. JW programmed several of the instruments used and reviewed the manuscript. BF designed several of the instruments used and reviewed the manuscript. MH is guarantor and designer of the study, contributed substantially to the interpretation of the analyses and the writing (Method section) and reviewing of the manuscript.

## Conflict of Interest Statement

The authors declare that the research was conducted in the absence of any commercial or financial relationships that could be construed as a potential conflict of interest.

## References

[B1] Albert D. Chein J. Steinberg L. (2013). The teenage brain: peer influences on adolescent decision making. *Curr. Dir. Psychol. Sci.* 22 114–120. 10.1177/0963721412471347 25544805 PMC4276317

[B2] Best J. R. Miller P. H. Naglieri J. A. (2011). Relations between executive function and academic achievement from ages 5 to 17 in a large, representative national sample. *Learn. Individ. Differ.* 21 327–336. 10.1016/j.lindif.2011.01.007 21845021 PMC3155246

[B3] Blakemore S.-J. Mills K. L. (2014). Is adolescence a sensitive period for sociocultural processing? *Annu. Rev. Psychol.* 65 187–207. 10.1146/annurev-psych-010213-115202 24016274

[B4] Briggs S. (2009). Risks and opportunities in adolescence: understanding adolescent mental health difficulties. *J. Soc. Work Pract.* 23 49–64. 10.1080/02650530902723316 29627907

[B5] Cappella E. Kim H. Y. Neal J. W. Jackson D. R. (2013). Classroom peer relationships and behavioral engagement in elementary school: the role of social network equity. *Commun. Psychol.* 52 367–379. 10.1007/s10464-013-9603-5 24081319 PMC4151566

[B6] Coté S. Tremblay R. E. Nagin D. S. Zoccolillo M. Vitaro F. (2002). Childhood behavioral profiles leading to adolescent conduct disorder: risk trajectories for boys and girls. *J. Am. Acad. Child Adolesc. Psychitry* 41 1086–1094. 10.1097/00004583-200209000-00009 12218430

[B7] Crone E. A. Dahl R. E. (2012). Understanding adolescence as a period of social-affective engagement and goal flexibility. *Nat. Rev. Neurosci.* 13 636–650. 10.1038/nrn3313 22903221

[B8] Crosnoe R. Johnson M. K. (2011). Research on adolescence in the twenty-first century. *Annu. Rev. Sociol.* 37 439–460. 10.1146/annurev-soc-081309-150008 29167597 PMC5695926

[B9] Davoudzadeh P. McTernan M. L. Grimm K. J. (2015). Early school readiness predictors of grade retention from kindergarten through eighth grade: a multilevel discrete-time survival analysis approach. *Early Child. Res. Q.* 32 183–192. 10.1016/j.ecresq.2015.04.005

[B10] De Laet S. Colpin H. Van Leeuwen K. Van den Noortgate W. Claes S. Janssens A. (2016). Transactional links between teacher-student relationships and adolescent rule-breaking behavior and behavioral school engagement: moderating role of a dopaminergic genetic profile score. *J. Youth Adolesc.* 45 1226–1244. 10.1007/510964-016-0466-6 27013478

[B11] Diamond A. (2013). Executive functions. *Annu. Rev. Psychol.* 64 135–168. 10.1146/annurev-psych-113011-143750 23020641 PMC4084861

[B12] Dosenbach N. U. F. Petersen S. E. Schlaggar B. L. (2013). The teenage brain: functional connectivity. *Curr. Dir. Psychol. Sci.* 22 101–107. 10.1177/0963721412474297

[B13] Dumontheil I. (2014). Development of abstract thinking during childhood and adolescence: the role of rostrolateral prefrontal cortex. *Dev. Cogn. Neurosci.* 10 57–76. 10.1016/j.dcn.2014.07.009 25173960 PMC6987955

[B14] Eiland L. Romeo R. D. (2013). Stress and the developing adolescent brain. *Neuroscience* 249 162–171. 10.1016/j.neuroscience.2012.10.048 23123920 PMC3601560

[B15] Epskamp S. Borsboom D. Fried E. (2016). Estimating psychological networks and their stability: a tutorial paper. *Behav. Res. Methods* 50 195–212. 10.3758/s13428-017-0862-1 28342071 PMC5809547

[B16] Fair D. A. Bathula D. Nikolaas M. A. Nigg J. T. (2012). Distinct neuropsychological subgroups in typically developing youth inform heterogeneity in children with ADHD. *Proc. Natl. Acad. Sci.* 109 6769–6774. 10.1073/pnas.1115365109 22474392 PMC3340031

[B17] Figner B. Mackinlay R. J. Wilkening F. Weber E. U. (2009). Affective and deliberative processes in risky choice: age differences in risk taking in the columbia card task. *J. Exp. Psychol. Learn. Mem. Cogn.* 35 709–730. 10.1037/a0014983 19379045

[B18] Fitzapatrick C. Archambault I. Janosz M. Pagani L. S. (2015). Early childhood working memory forecasts high school dropout risk. *Intelligence* 53 160–165. 10.1016/j.intell.2015.10.002

[B19] Foygel R. Drton M. (2010). Extended Bayesian information criteria for Gaussian graphical models. *Adv. Neural Inf. Process. Syst.* 23 604–612.

[B20] Friedman J. Hastie T. Tibshirani R. (2008). Sparse inverse covariance estimation with the graphical lasso. *Biostatistics* 9 432–441. 10.1093/biostatistics/kxm045 18079126 PMC3019769

[B21] Fruchterman T. M. Reingold E. M. (1991). Graph drawing by force-directed placement. *Softw. Pract. Exp.* 21 1129–1164. 10.1002/spe.4380211102

[B22] Goodman A. Lamping D. L. Ploubidis G. B. (2010). When to use broader internalising and externalising subscales instead of the hypothesised five subscales on the Strengths and Difficulties Questionnaire (SDQ): data from British parents. teachers and children. *J. Abnorm. Child Psychol.* 38 1179–1191. 10.1007/s10802-010-9434-x 20623175

[B23] Goodman R. (1997). The strengths and difficulties questionnaire: a research note. *J. Child Psychol. Psychiatry* 38 581–586. 10.1111/j.1469-7610.1997.tb01545.x 9255702

[B24] Goodman R. (2001). Psychometric properties of the strengths and difficulties questionnaire. *J. Am. Acad. Child Adolesc. Psychiatry* 40 1337–1345. 10.1097/00004583-200111000-00015 11699809

[B25] Guy S. C. Isquith P. K. Gioia G. A. (2004). *Behavior Rating Inventory of Executive Function - Self-Report Version.* Lutz, FL: Psychological Assessment Resources.

[B26] Harter S. (1985). *Manual for the Social Support Scale for Children.* Denver, CO: University of Denver.

[B27] Heeren A. McNally R. J. (2016). An integrative network approach to social anxiety disorder: the complex dynamic interplay among attentional bias for threat, attentional control, and symptoms. *J. Anxiety Disord.* 42 95–104. 10.1016/j.janxdis.2016.06.009 27395806

[B28] Hoorelbeke K. Marchetti I. De Schryver M. Koster E. H. W. (2016). The interplay between cognitive risk and resilience factors in remitted depression: a network analysis. *J. Affect. Disord.* 195 96–104. 10.1016/j.jad.2016.02.001 26878206

[B29] Huang G. C. Unger J. B. Soto D. Fujimoto K. Pentz M. A. Jordan-Marsh M. (2014). Peer influences : the impact of online and offline friendship networks on adolescent smoking and alcohol use. *J. Adolesc. Health* 54 508–514. 10.1016/j.jadohealth.2013.07.001 24012065 PMC4694047

[B30] Huizinga M. Dolan C. V. van der Molen M. W. (2006). Age-related change in executive function: developmental trends and a latent variable analysis. *Neuropsychologia* 44 2017–2036. 10.1016/j.neuropsychologia.2006.01.010 16527316

[B31] Huizinga M. Smidts D. P. (2012). *BRIEF Executieve Functies Gedragsvragenlijst: Handleiding.* Amsterdam: Hogrefe Uitgevers.

[B32] Ibrahim G. M. Morgan B. R. Vogan V. M. Leung R. C. Anagnostou E. Taylor M. J. (2016). Mapping the network of neuropsychological impairment in children with autism spectrum disorder: a graph theoretical analysis. *J. Autism Dev. Disord.* 46 3770–3777. 10.1007/s10803-016-2929-8 27696182

[B33] Jacobson L. A. Williford A. P. Pianta R. C. (2011). The role of executive function in children’s competent adjustment to middle school. *Child Neuropsychol.* 17 255–280. 10.1080/09297049.2010.535654 21246422 PMC4075458

[B34] Jaworska N. MacQueen G. (2015). Adolescence as a unique developmental period. *J. Psychiatry Neurosci.* 40 291–293. 10.1503/jpn.150268 26290063 PMC4543091

[B35] Jimmerson S. Egeland B. Sroufe L. A. Carlson B. (2000). A prospective longitudinal study of highschool dropouts examining multiple predictors across development. *J. Sch. Psychol.* 38 525–549. 10.1016/S0022-4405(00)00051-0

[B36] Jimmerson S. R. Ferguson P. (2007). A longitudinal study of grade retention: academic and behavioral outcomes of retained students through adolescence. *Sch. Psychol. Q.* 22 314–339. 10.1037/1045-3830.22.3.314

[B37] Juraska J. M. Willing J. (2017). Pubertal onset as a critical transition for neural development and cognition. *Brain Res.* 1654 87–94. 10.1016/j.brainres.2016.04.012 27060769 PMC5053848

[B38] Kellermann T. S. Bonilha L. Lin J. J. Hermann B. P. (2015). Mapping the landscape of cognitive development in children with epilepsy. *Cortex* 66 1–8. 10.1016/j.cortex.2015.02.001 25776901 PMC4405468

[B39] Konrad K. Firk C. Uhlhaas P. J. (2013). Brain development during adolescence. *Dtsch. Arztebl. Int.* 110 425–431. 10.3238/arztebl.2013.0425 23840287 PMC3705203

[B40] Kort W. Schittekatte M. Dekker P. H. Verhaeghe P. Compaan E. L. Bosmans M. (2005). *WISC-III NL. Handleiding en Verantwoording.* London: The Psychological Corporation.

[B41] Lewin-Bizan S. Lynch A. D. Fay K. Schmid K. McPherran C. Lerner J. V. (2010). Trajectories of positive and negative behaviors from early- to middle-adolescence. *J. Youth Adolesc.* 39 751–763. 10.1007/s10964-010-9532-7 20387107

[B42] Martel M. M. Levinson C. A. Langer J. K. Nigg J. T. (2016). A network analysis of developmental change in ADHD symptom structure from preschool to adulthood. *Clin. Psychol. Sci.* 4 988–1001. 10.1177/2167702615618664 28083448 PMC5222575

[B43] McNally R. J. (2016). Can network analysis transform psychopathology? *Behav. Res. Ther.* 86 95–104. 10.1016/j.brat.2016.06.006 27424882

[B44] Moffitt T. E. Arseneault L. Belsky D. Dickson N. Hancox R. J. Harrington H. (2011). A gradient of childhood self-control predicts health, wealth, and public safety. *Proc. Natl. Acad. Sci. U.S.A.* 108 2693–2698. 10.1073/pnas.1010076108 21262822 PMC3041102

[B45] Newman M. E. J. (2004). Detecting community structure in networks. *Eur. Phys. J. B* 38 321–330. 10.1140/epjb/e2004-00124-y 15244693

[B46] Newman M. E. J. (2006). Modularity and community structure in networks. *Proc. Natl. Acad. Sci. U.S.A.* 103 8577–8582. 10.1073/pnas.0601602103 16723398 PMC1482622

[B47] Prencipe A. Kesek A. Cohen J. Lamm C. Lewis M. D. Zelazo P. D. (2011). Development of hot and cool executive function during the transition to adolescence. *J. Exp. Child Psychol.* 108 621–637. 10.1016/j.jecp.2010.09.008 21044790

[B48] R Core Team. (2016). *R: A Language and Environment for Statistical Computing.* Vienna: R Foundation for Statistical Computing.

[B49] Ridderinkhof K. R. Van der Molen M. W. (1995). A psychophysiological analysis of developmental differences in the ability to resist interference. *Child Dev.* 66 1040–1056. 10.2307/1131797

[B50] Robbins S. B. Lauver K. Le H. Davis D. Langley R. Carlstrom A. (2004). Do psychosocial and study skill factors predict college outcomes ? A meta-analysis. *Psychol. Bull.* 130 261–288. 10.1037/0033-2909.130.2.261 14979772

[B51] Roid G. H. (2003). *Stanford-Binet Intelligence Scales.* Itasca, IL: Riverside Publishing.

[B52] Rubinov M. Sporns O. (2011). Weight-conserving characterization of complex functional brain networks. *Neuroimage* 56 2068–2079. 10.1016/j.neuroimage.2011.03.069 21459148

[B53] Russell J. D. Neill E. L. Carrion V. G. Weems C. F. (2017). The network structure of posttraumatic stress symptoms in children and adolescents exposed to disarters. *J. Am. Acad. Child Adolesc. Psychiatry* 56 669–677. 10.1016/j.jaac.2017.05.021 28735696

[B54] Silvers J. A. McRae K. Gabrieli J. D. Gross J. J. Remy K. A. Ochsner K. N. (2012). Age-related differences in emotional reactivity, regulation, and rejection sensitivity in adolescence. *Emotion* 12 1235–1247. 10.1037/a0028297 22642356 PMC4131311

[B55] Steinberg L. (2005). Cognitive and affective development in adolescence. *Trends Cogn. Sci.* 9 69–74. 10.1016/j.tics.2004.12.005 15668099

[B56] Steinberg L. Monahan K. C. (2007). Age differences in resistance to peer influence. *Dev. Psychol.* 43 1531–1543. 10.1037/0012-1649.43.6.1531 18020830 PMC2779518

[B57] van der Meer J. M. J. Lappenschaar M. G. A. Hartman C. A. Greven C. U. Buitelaar J. K. Rommelse N. N. J. (2017). Homogeneous combinations of ASD-ADHD traits and their cognitive and behavioral correlates in a population-based sample. *J. Atten. Disord.* 21 753–763. 10.1177/1087054714533194 24819924

[B58] van Widenfelt B. M. Goedhart A. W. Treffers P. D. A. Goodman R. (2003). Dutch version of the Strengths and Difficulties Questionnaire (SDQ). *Eur. Child Adolesc. Psychiatry* 12 281–289. 10.1007/s00787-003-0341-3 14689260

[B59] Vanhalst J. Luyckx K. Goossens L. (2014). Experiencing loneliness in adolescence: a matter of individual characteristics, negative peer experiences, or both? *Soc. Dev.* 23 100–118. 10.1111/sode.12019

[B60] Weil L. G. Fleming S. M. Dumontheil I. Kilford E. J. Weil R. S. Rees G. (2013). The development of metacognitive ability in adolescence. *Conscious. Cogn.* 22 264–271. 10.1016/j.concog.2013.01.004 23376348 PMC3719211

[B61] Zelazo P. D. Carlson S. M. (2012). Hot and cool executive function in childhood and adolescence: development and plasticity. *Child Dev. Perspect.* 6 354–360. 10.1111/j.1750-8606.2012.00246.x

